# Cry1Ab Treatment Has No Effects on Viability of Cultured Porcine Intestinal Cells, but Triggers Hsp70 Expression

**DOI:** 10.1371/journal.pone.0067079

**Published:** 2013-07-04

**Authors:** Angelika Bondzio, Ulrike Lodemann, Christoph Weise, Ralf Einspanier

**Affiliations:** 1 Institute of Veterinary Biochemistry, Freie Universität Berlin, Berlin, Germany; 2 Institute of Veterinary Physiology, Freie Universität Berlin, Berlin, Germany; 3 Institute of Chemistry and Biochemistry, Freie Universität Berlin, Berlin, Germany; Biological Research Centre of the Hungarian Academy of Sciences, Hungary

## Abstract

*In vitro* testing can contribute to reduce the risk that the use of genetically modified (GM) crops and their proteins show unintended toxic effects. Here we introduce a porcine intestinal cell culture (IPEC-J2) as appropriate *in vitro* model and tested the possible toxic potential of Cry1Ab protein, commonly expressed in GM-maize. For comprehensive risk assessment we used WST-1 conversion and ATP content as metabolic markers for proliferation, lactate dehydrogenase release as indicator for cells with compromised membrane and transepithelial electrical resistance as parameter indicating membrane barrier function. The results were compared to the effects of valinomycin, a potassium ionophore, known to induce cytotoxic effects in most mammalian cell types. Whereas no toxicity was observed after Cry1Ab treatment, valinomycin induced a decrease in IPEC-J2 viability. This was confirmed by dynamic monitoring of cellular responses. Additionally, two dimensional differential in-gel electrophoresis was performed. Only three proteins were differentially expressed. The functions of these proteins were associated with responses to stress. The up-regulation of heat shock protein Hsp70 was verified by Western blotting as well as by enzyme-linked immunosorbent assay and may be related to a protective function. These findings suggest that the combination of *in vitro* testing and proteomic analysis may serve as a promising tool for mechanism based safety assessment.

## Introduction

Microbial insecticides containing δ-endotoxins (Cry proteins) from *Bacillus thuringiensis* (Bt) have been used as an alternative to conventional chemical pesticides in agriculture for almost 60 years and recently as resource for insect-resistant genetically modified (GM) plants [1]. Currently, more than 90% of the feedstuffs for pigs contain genetic modified compounds [2] and the interest in GM crops is continuously increased because of higher agronomic productivity and more nutritious food without the use of pesticides. Since the introduction of GM crops many feeding trials focussed on issues related to consumer safety have been conducted in various animal species. In the majority of these studies no adverse effects have been detected. However, few studies found subtle histopathological changes and signs of hepatorenal toxicity in rats [3], [4] and altered immune responses in mice [5], fish [6] and pigs [7]. Thus, there is an on-going debate on the risk of GM consumption and a demand for additional evidence of GM food and feed safety [8]–[11].

Although *in vitro* testing of GM food and feed compounds is considered to be helpful to complement safety assessment programmes and has been encouraged by international scientific committees [10], only few data are available from cell culture experiments. The application of an *in* *vitro* cell culture system especially for preliminary screening of GM food has many advantages, e. g. sufficient results at low costs, high speed and less animal use [12]. Because of minor complexity of such cellular systems in comparison to the animal better conclusions can be drawn concerning specific mechanism of action. Moreover, mammalian cell cultures may allow scientists to reveal possible unintended side effects of novel proteins on non-target species. Thus, there is a growing interest in suitable *in vitro* screening systems possibly reflecting *in vivo* toxicity of food ingredients.

Liver and kidney are considered as the two major target organs of detoxification. Therefore cell cultures derived from these organs are in the focus of risk assessment. For example, a slight but not statistically significant increase of LDH release after 48 h exposure to Cry1Ab was observed on bovine hepatocytes [13]. Moreover Bt toxins have been tested on human embryonic kidney cells [14]. Time- and dose dependent effects of relatively high concentrations of Cry1Ab on viability of HEK293 cells, respiration inhibition and plasma membrane alterations, were detected. In addition, cell cultures from the gastrointestinal tract (GIT) are of particular interest in comprehensive risk assessment. Cell cultures of the digestive system are clearly superior to the use of any other cell types, because the GIT represents the first barrier for exogenous food and the primary portal and absorption side. Notably, since very low amounts of full-size and fragmented Cry1Ab protein have been detected in the GIT digesta [15]) in the rumen [16], [17] and in the GIT of pigs [18], such intestinal cell culture systems are also in the focus of GM safety research. From the results on brush-border membrane vesicles (BBMVs) it was concluded, that Cry1Ab may not impair the membrane integrity or permeability of mammalian intestinal epithelial cells [19]. In contrast, our previous results on perfused rumen epithelial cells suggest that at sufficiently high concentrations spontaneous insertion of Cry1Ab into the membrane of these cells occurs [20]. Nevertheless, we found no adverse effects on viability of cultured rumen epithelial cells [21]. Consequently, there is a need for additional data in comprehensive risk assessment regarding Cry1Ab on suitable *in vitr*o systems.

The aim of this study was to identify possible effects of Cry1Ab on porcine intestinal cells. The used IPEC-J2 cell line is well characterized [22] and represents a convenient intestinal functional cell model [23], that reflects the *in vivo* situation as faithfully as possible. A novel electronic cell sensor array technology, the real-time cell analysis (RTCA) system was used for dynamic monitoring of cellular events of Cry1Ab response. In addition, different viability parameters as the ATP level, the WST-1 conversion, the release of lactate dehydrogenase (LDH) and the transepithelial electrical resistance (TEER) were used to get a comprehensive data set to complete the understanding whether Cry1Ab affects the viability of gastrointestinal cells. Furthermore, the two dimensional differential in gel electrophoresis (2D-DIGE) combined with mass spectrometry was employed to supplement the viability approaches to better characterize the possible interactions of Cry1Ab at the molecular and cellular levels. The identified changes of Hsp70 level caused by Cry1Ab treatment were further verified by Western blotting and enzyme-linked immunosorbent assay (ELISA) using specific antibodies.

## Materials and Methods

### Cell Culture

The IPEC-J2 cell line is a non-transformed intestinal cell line originally derived from jejunal epithelia, isolated from a neonatal, unsuckled piglet [22]. Cells were grown in Dulbecco’s modified eagle medium (DMEM)/Ham’s F-12 (1∶1) supplemented with 10% fetal calf serum (Biochrom, Germany) and maintained in an athmosphere of 5% CO_2._ For viability measurements IPEC-J2 cells were plated at 0.5×10^4^ cells per well in 96-well plates, for Hsp detection cells were seeded at 0.5×10^4^ cells per well in 24-well plates. After 24 h subconfluent cells were exposed to different Cry1Ab concentrations for indicated time intervals. As a control, cells were kept under same conditions without Cry1Ab. For proteomic profiling IPEC-J2 cells of different passages (35–40) were grown in 75 cm^2^ flasks. After reaching 80% confluence, the cells were washed and the medium was replaced. The cells were exposed to 1 µg/µl Cry1Ab for 24 h. For the control cells, the medium without Cry1Ab was used.

### Purification of Cry1Ab Protein

Cry1Ab was derived from the BMBF project 01K0-31P2614, Germany. The protoxin Cry1Ab was prepared with a standardised procedure from *E. coli HB101/pMP* as described by Nguyen [24]. Protoxins were activated by trypsinization and further purified, yielding a toxin comprising a primary structure similar to toxins present in transgenic plants. Size and purity were confirmed by sodium dodecyl sulphate-polyacrylamide gel electrophoresis (SDS-PAGE) [24]. Finally, Cry1Ab was stored in 0.5 mM CAPS buffer (pH 10.5). The toxicity was determined by a bioassay on susceptible *Ostrinia nubilalis* larvae and the LC50 value (toxin concentration at which 50% of the test animals die) was 50 ng/cm^2^ for surface application [25]. The Cry1Ab toxin was stable in our cell culture system for at least 48 hours, as tested by immunoblotting using anti-Cry1Ab mouse monoclonal antibody kindly provided by Dr. Wal (INRA, France).

### Evaluation of Cell Viability

Cell proliferation was detected using the Cell Proliferation Reagent WST-1 (Roche). The quantitation of ATP was used as a further indicator of viable cells measured with CellTiter-Glo Luminescent Cell Viability Assay (Promega). The released LDH was determined using the CytoTox-ONE™ Homogeneous Membrane Integrity Assay (Promega), a fluorimetric method for measuring the release of LDH from cells with damaged membrane. These methods were performed according to the manufacturer's instructions and have been described in detail by our group in a previous publication [21].

IPEC-J2 cells were treated for 24 h respectively for 48 h with 500 ng/ml and 1 µg/ml Cry1Ab. For all viability assays valinomycin (500 ng/ml, ∼0.556 µg/ml respectively) was used as cell damaging control.

### Real-time Cell Analysis

The xCELLigence system (Roche Applied Science and ACEA Biosciences) was used to evaluate cell survival after Cry1Ab treatment. The real-time cell analysis was performed under cell culture conditions (37°C, 5% CO_2_, 95% humidity) on the E-plate 96 that differs from standard 96-well microtiter plates vastly with its incorporated gold cell sensor arrays in the bottom. The impedance of the sensor electrodes was measured to allow monitoring the physiological behaviour of the cells on the electrodes. In the presence of cells, cells attached to the electrode sensor surfaces will lead to an increase in impedancehttp://www.sciencedirect.com/science/article/pii/S0109564109002887 - bbib30#bbib30. Thus, the Real-time xCelligence impedance measurement correlates with changes in cell proliferation, viability, and cytotoxicity. The xCELLigence system was used according to the instructions of the supplier (Roche Applied Science and ACEA Biosciences). First, the optimal seeding concentration was determined. After seeding the respective optimal number of cells (10,000/well) in 100 µl medium to the wells of the E-plate 96, the physiological state of the IPEC-J2 cells was monitored every 30 min by the xCELLigence system. After 4 h, when cells were in log growth phase, the IPECJ-2 cells were exposed to 50 µl of medium containing 1 µg/ml Cry1Ab or 500 nM valinomycin, respectively. Controls received medium only, or medium plus ethanol, respectively. Each experiment was run for 24 h.

### Transepithelial Electrical Resistance (TEER) Measurement

For TEER measurements, the cells were seeded on clear polyester membrane cell culture inserts (Snapwell^®^, 12 mm diameter, 1.12 cm^2^ area, 0.4 µm pore size; Corning B.V., Schiphol-Rijk, Netherlands) at a density of 10^5^ cells/1.12 cm^2^ and were allowed to differentiate for 7–10 days. TEER measurements were performed by using a Millicell-ERS (Electrical Resistance System; Millipore GmbH, Schwalbach, Germany).

Cry1Ab was added when absolute TEER reached values at least of 2500 Ω · cm^2^ indicating a confluent monolayer [22], [26]. Increasing doses of Cry1Ab (0.1, 0.5, 1 µg/ml) or valinomycin (500 nM) were added at the apical side. The TEER was measured after 24 and 48 h of treatment.

### Two-dimensional Differential in Gel Electrophoresis (2-D DIGE) Analysis

Extracts of total cell protein were prepared 24 h after exposure to 1 µg/ml Cry1Ab. Cultured cells were washed twice with ice-cold PBS, and lysed in lysis buffer (20 mM HEPES pH 7.8; 0.35 mM NaCl; 20% glycerol; 1% NP 40; 1 mM MgCl_2_; 1 mM dithiothreitol; 1 mM phenylmethylsulfonyl fluoride; 0.5 mM EDTA; 0.5 mM EGTA; 0.5 mg/ml aprotinin and 1 mg/ml leupeptin) for 30 min at 4°C. Cell lysates were centrifuged at 16,000 g for 5 min, and the supernatants were used for 2-D DIGE.

For 2-D DIGE, a pool consisting of equal amounts of each cell extract was prepared as an internal standard. Thus every protein from every sample was represented in this standard on all gels. This internal standard was always labeled with Cy2 and run together with individual samples, from control and Cry1Ab treated cells, labeled with other CyDyes (Cy3 or Cy5, GE Healthcare). Each sample (60 µg) was labeled with 480 pmol appropriate CyDye following the manufacturer’s protocol. The total volume of each labeled protein sample (3×60 µg) was adjusted to 300 µl with DeStreak^-^™ Rehydration Solution (GE Healthcare). Samples were applied to isoelectric focusing (IEF) strips (3–10 pH range, nonlinear, 17 cm; BIO-RAD), which were overlaid with 2.5 ml DryStrip Cover Fluid (GE Healthcare) and equilibrated for 14 h at 50 V and isoelectrically focused at 1 h 200 V, 1 h 500 V, and finally at 10.000 V for 7 h by using a Protean IEF cell (BIO-RAD). Thereafter, the strips were equilibrated for 15 min with gentle shaking in 50 mM Tris-HCl (pH 8.8) containing 6 M urea, 4% SDS, 65 mM dithiotreitol (DTT), 30% glycerol, and 0.02% bromophenol blue; 135 mM iodoacetamide was added to the second equilibration solution instead of DTT, and the strips were further incubated for 15 min in this solution. Subsequently, strips were loaded onto 12% acrylamide vertical gels. SDS-polyacrylamide gel electrophoresis (SDS–PAGE) for the second dimension was carried out in an ETTAN DALT *six* electrophoresis unit (GE Healthcare), first at 0.2 W per gel for 1 h and thereafter at 2 W per gel for 18 h.

### Image Acquisition and Analysis

Protein spots were visualized by using the Typhoon 9400 laser imager (GE Healthcare) choosing the appropriate wavelength for each Cy dye (Cy2 = 520nm; Cy3 = 580 nm; Cy5 = 670 nm) at a resolution of 100 µm, were cropped and imported into DeCyder V.7.0 software (GE Healthcare). During spot detection by a co-detection algorithm in the software, the estimated number of spots were set at 2500, and the exclude filter was set a slope >1.7 and area <200. Only those proteins that appeared to be differentially expressed in five gels were considered as differentially regulated. The DeCyder differential in the gel analysis (DIA) modul was used to process the images from a single gel and enables the pair-wise comparison of each control and Cry1Ab-treated sample to the pooled internal standard. The abundance of each protein spot was determined as a ratio to its corresponding spot present in the internal standard on the same gel. The DeCyder biological variation analysis (BVA) module was used to standardize the ratios across the gels accounting for the differences in the internal standard.

### Protein Identification via In-gel Digestion and Mass Spectrometry

Changes of protein expression in response to Cry1Ab treatment detected by 2-D DIGE analysis were matched with silver-stained protein patterns (400 µg protein), and the three selected spots that showed at least a 1.3-fold change in protein expression were excised from the gel. Protein spots were in-gel digested by trypsin (Promega, Germany) and proteins were further analyzed mass-spectrometrically. A nanoLC-ESI-MS/MS analysis was performed by Proteome Factory Berlin (Proteome Factory AG, Germany) using an Agilent 1100 nanoLC system (Agilent, Germany), PicoTip emitter (New Objective, USA) and an Esquire 3000 plus ion trap MS (Bruker, Germany). For protein identification MS/MS ion search of Mascot search engine (Matrix Science, England) and nr protein database (National Center for Biotechnology Information, Bethesda, USA) were used. Ion charge in search parameters for ions from ESI-MS/MS data acquisition was set to “1+, 2+ or 3+” according to the instrument's and method's common charge state distribution. Alternatively, protein digests were analyzed by matrix-assisted laser desorption ionization-time of flight mass spectrometry (MALDI-TOF-MS) using an Ultraflex-II TOF/TOF instrument (Bruker Daltonics, Bremen, Germany) equipped with a Smart beam™ laser. Peptide mass fingerprints were recorded in the reflector mode using α-cyano-4-hydroxycinnamic acid (CHCA) as the matrix.

### Detection of Hsp70

Hsp70 concentration in IPEC-J2 extracts was quantified by an ELISA (StressXpress Hsp70 ELISA; Stressgen Biotechnologies, Victoria, BC, Canada) in accordance with the manufacturer’s instructions. The Hsp70 values were normalized to the protein concentration.

For Western blot analysis cells were lysed in RIPA buffer containing a protease inhibitor mixture (Merck Biosciences). Sample proteins (20 µg/lane) and a prestained protein-weight marker (Bio-Rad Laboratories GmbH) were resolved by SDS-PAGE (12% polyacrylamide gels) and transferred onto nitrocellulose membranes in Tris-glycine buffer with 20% (v/v) methanol. The membrane was saturated with 5% (w/v) non-fat milk powder (Roth) prepared with phosphate-buffered saline containing 0,1% Tween20 (PBST) for 1 h at room temperature, then incubated with anti-Hsp70 mouse monoclonal antibody (Stressgen) overnight at 4°C. After several washings with PBST the membranes were incubated with a 1∶20000 dilution of an anti-rabbit IgG horseradish peroxidase-conjugated secondary antibody (GE Healthcare). Bound antibodies were detected by using an enhanced chemiluminescence system ECL Advance according to the manufacturer’s instructions. Membranes were again incubated with glyceraldehyde 3-phosphate dehydrogenase (GAPDH) mouse monoclonal antibody (Abcam) to normalize the results. The protein concentration of all extracts used in immunoassays was determined with 2-D Quant Kit (GE Healthcare).

## Results

### IPEC-J2 Cell Responsiveness to Cry1Ab using Different Parameters of Viability

**Figure 1:**
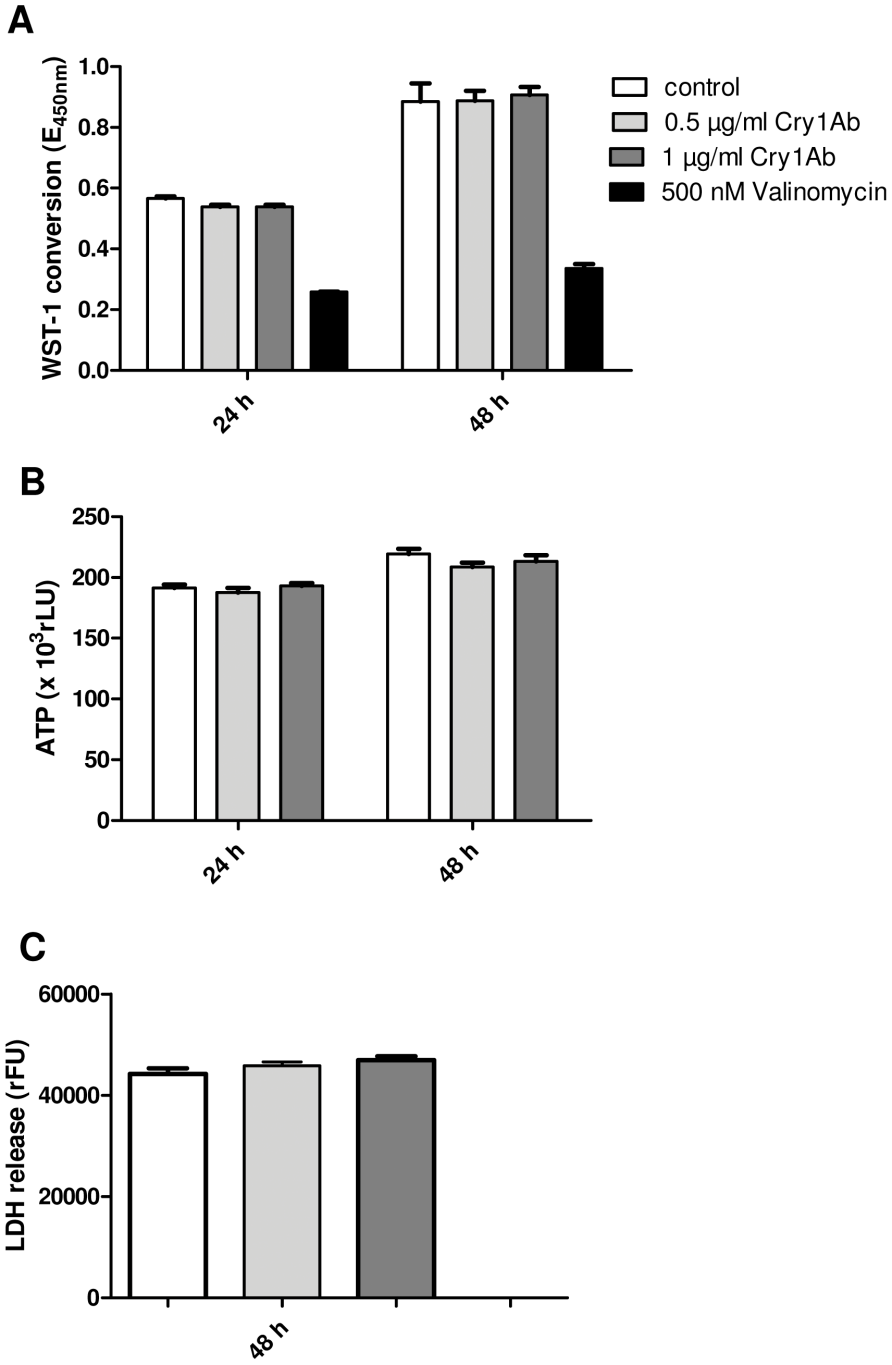
Effect of Cry1Ab on viability of IPEC-J2 cells. IPEC-J2 cells were incubated with indicated concentrations of Cry1Ab for 24 h and 48 h, respectively. Data are from one representative experiment out of 2 performed. (A) The Cell Proliferation Reagent WST-1 (Roche) was used to measure mitochondrial dehydrogenase activity of viable cells. Valinomycin was used as cell damaging control. Bars represent the mean +/−S.D. of 5 replicate wells. (B) The CellTiter-Glo Luminescent Cell Viability Assay (Promega) was used to estimate the number of viable metabolically active cells based on quantitation of ATP. Bars represent the mean +/−S.D. of 5 replicate wells. (C) The CytoTox-ONE™ Homogenous Membrane Integrity Assay (Promega) was used for estimating the number of dead cells by release of LDH from damaged cells. Bars represent the mean +/−S.D. of 12 replicate wells.

**Figure 2:**
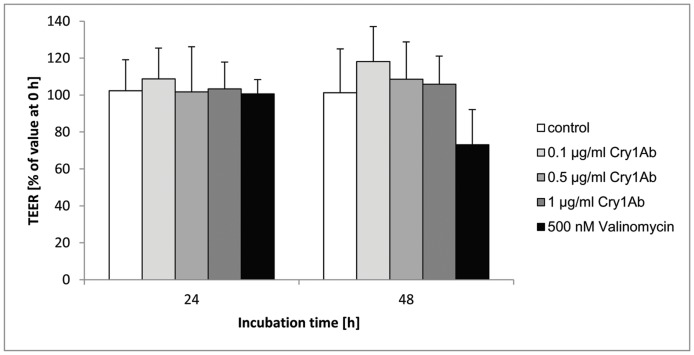
Effect of Cry1Ab on transepithelial electrical resistance (TEER) in porcine intestinal epithelial monolayers. TEER of IPEC-J2 cell monolayers was measured 24 h and 48 h after application of increasing doses of Cry1Ab (0.1, 0.5, 1 µg/ml) or valinomycin (500 nM) at the apical side [means ± standard deviation; % of initial value t  = 0 h]; n = 3 independent experiments (2–3 cell culture inserts per treatment group per experiment).

By investigating different biochemical and physiological parameters of viability it was demonstrated that isolated Cry1Ab protein does not influence the viability of porcine intestinal cells. Even when using non-physiologically high concentrations of Cry1Ab (500 ng/ml-1 µg/ml), no significant effects were found. LDH release, WST-1 conversion, ATP content and TEER were used to evaluate cytotoxicity of Cry1Ab, one of the expressed proteins in transgene Bt-maize. The mitochondrial dehydrogenase activity indicated by WST-1 conversion, as well as the ATP level reflecting proliferation and metabolism were not significantly different to untreated cells within 48 hours (Fig. 1A and 1B). Furthermore the LDH-release, indicating membrane permeability alterations, was not changed (Fig. 1C). The functional integrity of polarized IPEC-J2 monolayers was assessed by TEER measurements. Figure 2 shows TEER determined at 2 different time points during 48 h of Cry1Ab treatment. TEER was not significantly different between the Cry1Ab treated cell monolayers and the controls. We applied valinomycin as test control, as used in our previously published experiments on rumen epithelial cells [21]. Valinomycin as potassium ionophore is known to induce permeability changes like that of Cry1Ab in target cells [27]. Whereas no toxicity of Cry1Ab was observed in our experiments, valinomycin causes a response of all viability parameters (Fig. 1A–C). After 48 h the activity of dehydrogenases decreased at 500 nM valinomycin by 63%, accordingly the ATP content decreased by 47% and the LDH release increased by 42%, revealing membrane alterations.

### Real-time Monitoring of Cry1Ab Response

**Figure 3:**
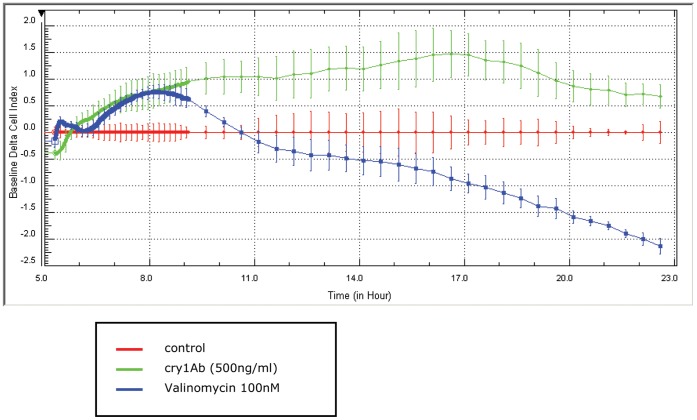
Dynamic monitoring of IPEC-J2 cell response after treatment with Cry1Ab and valinomycin using the xCELLigence system. IPEC-J2 cells of 10,000 cells/well in E-plate96 were observed during 24 h. Data points represent mean values +/− SEM (n = 5 wells).

Furthermore, the real-time monitoring of nonconfluent IPEC-J2 cells clearly indicates no toxic effect of Cry1Ab. Meanwhile valinomycin leads to a short increase of the delta cell index which later declines continuously below that of the control (Fig. 3). For Cry1Ab treatment an increase of the delta cell index was observed during the first hours after treatment. Thereafter it returns to the baseline of the control cells (after 24 h) indicating a mild short term and reversible effect of Cry1Ab.

### Protein Expression Changes in Response to Cry1Ab

**Figure 4:**
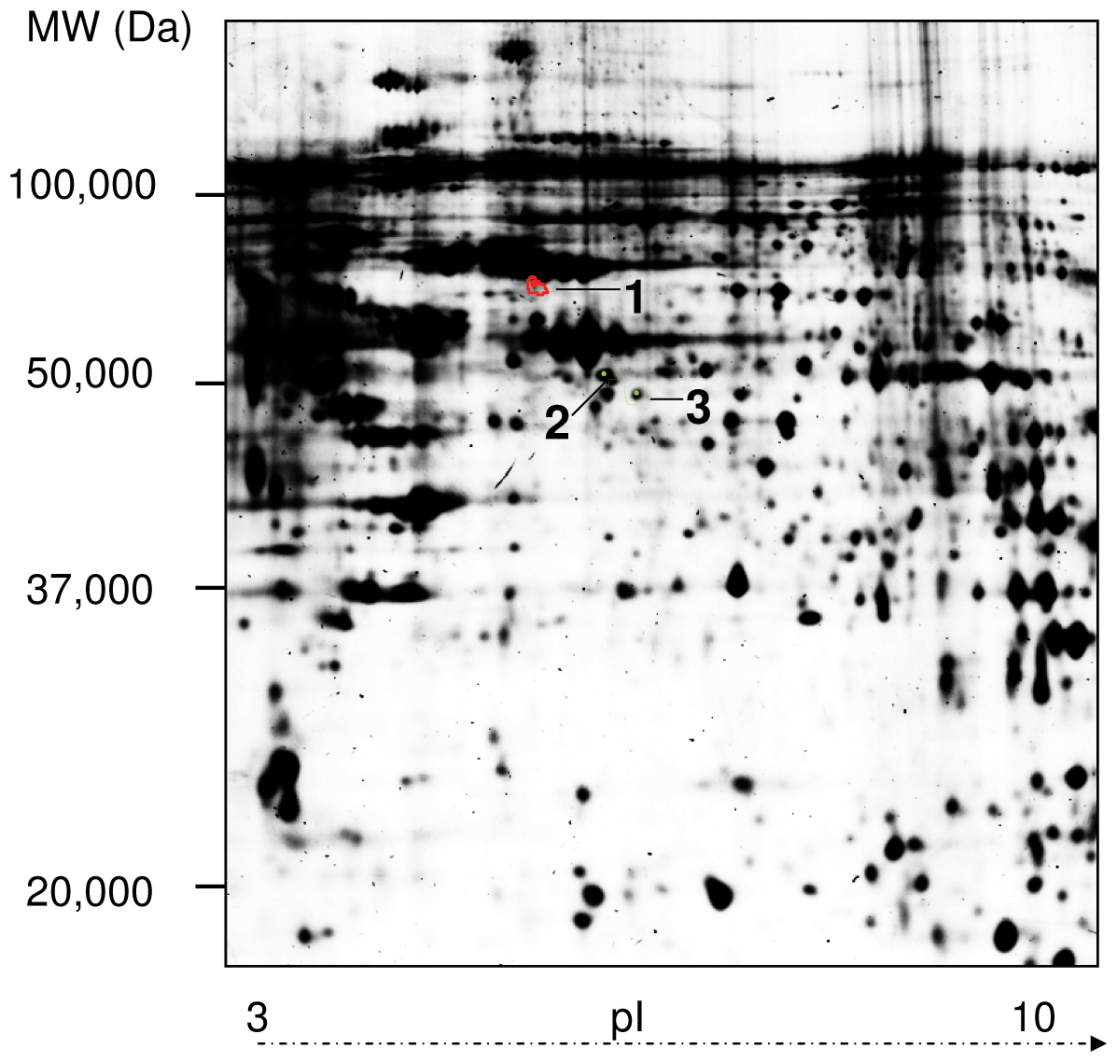
Proteomic profiling of IPEC-J2 cells in response to Cry1Ab treatment as revealed by 2D DIGE analysis. Image shows one representative spot map of Cry1Ab treated IPEC-J2 cell extracts (n = 5) indicating spot boundaries of proteins, whose expression level is increased (red) or decreased (green) in comparison with the corresponding untreated controls (only medium) (P<0.05) as revealed by Decyder V.7.0 software. Spots marked with a number, correlating to the identified proteins in Table1.

To analyze global protein expression changes induced by Cry1Ab a quantitative proteome analysis of porcine intestinal cells (IPEC-J2) was performed. After 24 h incubation with a non-physiological high concentration of Cry1Ab (1 µg/ml) protein extracts were prepared as described. Five biological replicates per treatment were reverse labelled by Cy3 and Cy5 and run together with the Cy2 labelled internal standard giving a total of 15 images. Using DeCyder Software analysis about 2500 spots were detected in each gel and quantified, normalized and inter-gel-matched.. Protein expression changes were considered as significant when their quantity decreased or increased by at least 1.3-fold (student’s T-test, p≤0.05). Notably, Cry1Ab treatment caused only few modifications in protein expression profiles, only three proteins out of >2000 were differentially expressed (Table 1), one protein (heat-shock protein 70) was more abundant and two proteins (cytokeratin 8 and heterogeneous nuclear ribonucleoprotein) were less abundant. The location of these spots is indicated in Figure 4 and the protein name, NCBI database accession number, Mascot score and statistics for the three identified proteins are given in Table 1.

**Table 1:** List of identified differentially expressed proteins in Cry1Ab treated IPEC-J2 cells.

Identified Protein	No.	pI^a^	Mass (Da)^a^	Score	Accession no.^b^	Average ratio^c^	No. of matched Peptide (% PC)^d^
Heat shock 70 kD protein	1	5.8	71065	102	gi 178056524	+1.31	20 (36)
Keratin, type II, cytoskeletal 8	2	5.7	54394	236	gi 227430407	−1.31	25 (48)
Heterologenous nuclear ribonukleoprotein H2-like	3	6.1	49073	221	gi 335310367	−1.37	23 (47)

a) Theoretical isoelectric point (pI).

b) Accession number in NCBI database.

c) Average ratio (Cry1Ab treated/non-treated control) indicates the value derived from the normalized spot volume standardized against the intra-gel standard provided by DeCyder software analysis.

d) Peptide coverage, the amount of the identified proteins that the peptides covered.

**Figure 5:**
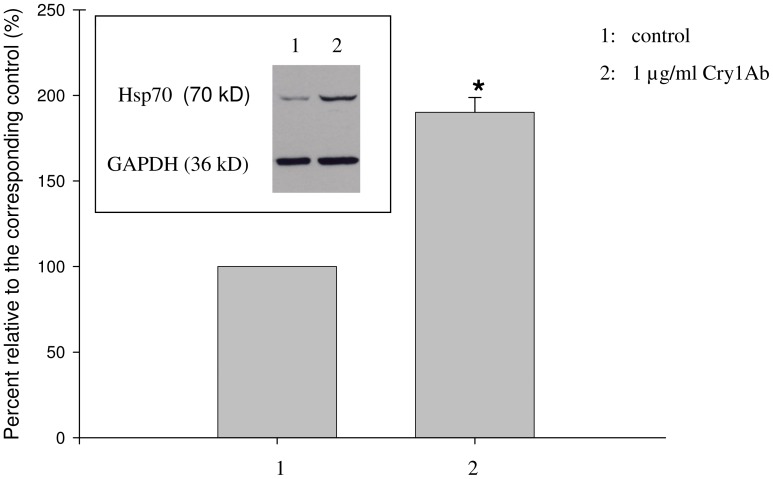
Increase of Hsp70 protein expression in Cry1Ab-treated IPEC-J2 cells. IPEC-J2 cells were treated with Cry1Ab for 24 hours and the Hsp70 protein was determined by ELISA and Western blot (insert). For ELISA Hsp70 is normalized to µg protein extract used in the assay. The normalized data are represented as mean relative to the control. Data represent the mean +/− SD; n = 5 different experiments; (*) results are significantly different as compared with untreated controls (P<0.05). For Western blotting the membranes were additionally incubated with glyceraldehyde 3-phosphate dehydrogenase (GAPDH) mouse monoclonal antibody (Abcam), which serves as control for protein expression.

To validate the DIGE/MS results the selected up-regulated stress response biomarker Hsp70 was further quantified at the protein level. The induction of Hsp70 was determined by ELISA and verified by Western blotting (Fig. 5). Confirming the proteomic profile, Fig. 5 indicates that Cry1Ab induces Hsp70 expression of porcine intestinal cells. Cultured IPEC–J2 cells constitutively express Hsp70. Treatment of these cells with 1 µg/ml Cry1Ab lead to induction of Hsp70 protein. As indicated in Fig. 5 protein extracts from cells of 5 different passages were analyzed by ELISA and show significant induction of Hsp70 (1.8-2 fold). The maximum level was reached within 24 h. Differences between Hsp70 levels of Cry1Ab treated and non-treated control cells were also confirmed by Western blotting (Fig. 5).

## Discussion

The increasing presence of GM food and feed on the market has provoked a strong demand for a comprehensive risk assessment by use of 90-day rat feeding studies [28]. Alternative *in vitro* cell culture assays may contribute to risk assessment yielding supplementary information on cellular physiological and biochemical mode of action of novel feed and food compounds.

In the present study we investigated whether Cry1Ab has any effect on the viability of porcine intestinal cells (IPEC-J2). IPEC-J2 cells were selected for this study because this cell line maintains differentiated characteristics and exhibits strong similarities to primary intestinal epithelial cells [22]. Therefore it represents a potentially more suitable model than most commonly used transformed cell lines. It is also of note that this cell line may represent an appropriate *in vitro* model with obvious homology to humans for investigating gastro-intestinal reactions. Measurement of multiple parameters of viability provides a better understanding of the physiological state of the cells [12], [21], [29]. Therefore we performed different endpoint assays. Particularly, the WST-1 conversion and the ATP content indicating the energy metabolism and the LDH release reflecting the membrane integrity of the cells were used in order to get a more comprehensive picture of the impact of Cry1Ab on IPEC-J2 cells. Moreover, we used TEER as functional parameter on differentiated IPEC-J2 cells grown on collagen-coated membranes in order to characterize the barrier function. Based on the results of these different parameters of cell viability we were able to exclude a short-term toxic effect of Cry1Ab on cultured IPEC-J2 cells.

Furthermore, we used the xCELLigence system for dynamic monitoring of immediate cell response by use of cellular impedance measurements [30], [31]. Such real-time monitoring allows label-free assessment of cell proliferation and enables continuous information about any changes in morphology and growth. Particularly, it is also advantageous that small and fast changes can be detected using this method [32], [33]. For validation of real-time monitoring to measure cell fitness we used valinomycin as apoptosis inducer [34], just as used for assay control in our recent study on ruminal cells [21]. While for Cry1Ab a receptor dependent specific toxicity to insect midgut epithelial cells is discussed [35]–[39], it is generally accepted that valinomycin acts independently of receptor(s) as a potassium ionophore leading to ion leakage and inducing cytotoxic effects *in vivo* and *in vitro* [40]–[42]. Therefore we used valinomycin as cell-damaging control and could demonstrate that this K^+^ ionophore, in contrast to Cry1Ab, exerts cytotoxic effects. The obvious changes in cell index are consistent with reduction in cell viability and with the observed decrease in TEER after 48 h. Consistent with the results of our previous study [21]. The activity of the mitochondrial dehydrogenases indicated by WST-1 conversion is the most sensitive parameter of viability. However, regarding possible Cry1Ab effects, no cytotoxic influence was found neither by the use of the endpoint assays nor by real-time monitoring. Interestingly, we observed a temporary increase in delta cell index of Cry1Ab treated cells in comparison to control cells during the first 17 hours. This effect may be associated with stress induced remodelling of cytoskeleton, a suggestion that is further supported by our proteomic analysis. However, this effect was reversible and for the precise interpretation of these real-time data further studies should be performed by simultaneous monitoring of cellular morphological changes.

In the present *in vitro* approach we have also included a molecular proteomic profiling technique (2-DE) for Cry1Ab target profiling and mechanism-based safety evaluation. Only few proteins were found to be significantly modulated. The identified proteins are multifunctional and involved in important cellular processes as discussed in the following section.

Heat shock proteins like Hsp70 are cellular chaperone proteins that are required for essential cellular functions, such as, protein folding and assembly or reassembly. Among the different families of these proteins, the Hsp70-family consists of at least eight highly homologous members (reviewed by Tavaria et al. [43] and Daugaard et al. [44]). It has been reported, that the expression of the two major members, the constitutive Hsp70c and the inducible Hsp70 expression could be enhanced in response to different stress conditions [45]–[47]. Previously these stress proteins have been used to monitor the impact of environmental factors on various animal species (reviewed by Mukhopadhyay et al. [48]), including pig [49] as well as in *in-vitro* models [50]. Furthermore, Hsp70 is known to inhibit the aggregation of nascent or misfolded proteins to regulate protein degradation and to help in translocation of proteins between different cellular compartments (reviewed by Hartl et al. [51] and Arya et al. [52]). In addition to its chaperoning function the Hsp70 protein family is required for cell survival and therefore closely linked to cytoprotection by conferring a protection of stressed cells. This may be related to the ability of Hsp70 to prevent protein modifications [53], or it may be explained by the ability of Hsp70 to inhibit stress-induced apoptosis [54], [55]. Furthermore it has been shown that the induction of Hsps induced by either modest, physiologically relevant increase in temperature or by glutamine supplementation protects intestinal epithelial cells against injury and must not be considered as adverse effect per se [50], [56]–[57]. Hsp70 protects the cells via multiple mechanisms, which include prevention of caspase-dependent apoptosis in different cell types, including intestinal cells [50], [56]–[59]. In our earlier study, neither cytotoxic effects nor signs of apoptosis could be detected in rumen epithelial cells even at the same concentration of Cry1Ab [21]. Here we did not test for apoptosis, but in the line with our previously published paper we could not find any evidence for Cry1Ab mediated cytotoxicity. Thus we can speculate, that the induction of Hsp70 in response to Cry1Ab in pig intestinal cells as found in our proteomic analysis and further demonstrated by Western blotting and ELISA may represent an adaptive response for the maintaining of cellular homeostasis under stress. As further shown by the proteomic profile in response to Cry1Ab IPEC-J2 cells down-regulate cytokeratin 8 expression. The cellular consequences of this altered cytokeratin expression are unknown. Interestingly molecular chaperones perform cooperating roles in the regulation of cytoskeleton function. It is known, that members of Hsp family can interact with intermediate filaments and the cytoskeleton. Particularly, Hsp70 immuno-precipitates with keratins 8 or 18 were found in several organs, including the digestive tract. However, the nature of this relationship still remains uncertain (reviewed by Liang and MacRae [60]. Generally, keratin functions are facilitated by dynamic association with other proteins and by posttranslational modifications like glycosylation [61]. The molecular mechanisms governing keratin assembly or disassembly are largely unknown [62] but several functions are discussed, including protection of epithelial tissue from injury [63].

Furthermore, the heterogeneous nuclear ribonucleoprotein (HnRNP) H2-like was differentially expressed under Cry1Ab treatment. HnRNPs are a family of abundant nuclear proteins with a wide range of molecular weights, which are believed to be involved in pre-mRNA packaging and processing [64]. In fact, these proteins have a variety of molecular partners (such as e.g. Hsps) with multiple functions in signal transduction (reviewed by Bomsztyk et al. [65] and [66], their special function in porcine intestinal cells, however, remains speculative.

In summary, the present study shows that cultured porcine gastrointestinal cells can tolerate Cry1Ab even in a dose range that greatly exceeds any amount that may accumulate in the gastrointestinal tract of pigs. No influence on viability of IPEC-J2 cells was found using a screening with different assays including real-time monitoring of cell viability. Nevertheless, consistent with a previously published *in vivo* study in fish [6], our proteomic data at 24 h were indicative of a mild stress response to Cry1Ab. Further studies, particularly long-term investigations are needed to determine whether increased Hsp70 expression is only a transient short-term adaptive response to Cry1Ab or may be the cause of further unintended side effects of this protein.
